# Assessing the Utility of the Aspartate Aminotransferase to Platelet Ratio Index (APRI) as a Noninvasive Indicator for Liver Cirrhosis

**DOI:** 10.7759/cureus.59680

**Published:** 2024-05-05

**Authors:** Siva Reddy, Sachin Agrawal, Harshitha Reddy, Sunil Kumar, Rushikesh H Dhondge, Sourya Acharya, Manjeet Kothari, Maimoona Khan, Chaitanya Kumar Javvaji

**Affiliations:** 1 Internal Medicine, Jawaharlal Nehru Medical College, Datta Meghe Institute of Higher Education and Research, Wardha, IND; 2 Pediatrics, Jawaharlal Nehru Medical College, Datta Meghe Institute of Higher Education and Research, Wardha, IND

**Keywords:** fibrosis, liver cirrhosis, aspartate aminotransferase to platelet ratio index (apri score), cases, life prognosis, cirrhosis mortality

## Abstract

Background

Of liver-related disorders, cirrhosis is currently the leading cause of death and has become a significant global public health concern. Aspartate aminotransferase to platelet ratio index (APRI), a newer prognostic modality, is a very effective noninvasive diagnostic for identifying advanced liver fibrosis.

Methods

A prospective observational study was conducted among individuals with liver disease, 100 cases and 100 controls for two years. All the sociodemographic details, clinical features of the patients, and clinical findings such as prothrombin time (PT), liver function tests, kidney function tests, and total blood count were recorded using a pretested semi-structured questionnaire.

Results

According to our survey results, 48% of the participants were between the ages of 40 and 60. Regarding aPTT (activated partial thromboplastin time) and liver function test characteristics (serum glutamic-oxaloacetic transaminase(SGOT), serum glutamic pyruvic transaminase (SGPT)), we showed a substantial difference between the patients and controls. Regarding the APRI distribution, we also found a statistically significant variation between the research groups. When we compared the validity of APRI scores in diagnosing cirrhosis, we discovered that the ideal cutoff value of APRI was determined to be 3.99, with sensitivity, specificity, positive predictive value (PPV), and negative predictive value (NPV) of 33%, 86%, 70%, and 56%, respectively. The area under the receiver operating characteristic (ROC) curve for APRI in detecting cirrhosis was also 0.693.

Conclusion

Thus, our study results conclude that APRI is a crucial noninvasive prognostic tool that can be utilized to prognostize liver cirrhosis.

## Introduction

Of liver-related disorders, cirrhosis is currently the leading cause of death and has become a significant global public health concern. It is characterized by defective microcirculation, liver parenchymal and anatomical defects, change in hepatic architecture, distortion, and regenerative nodular formation, resulting in progressive fibrosis. When it has reached its final stages of progression, it becomes irreversible, and the only treatment often left after that would be liver transplantation. In contrast, in its earlier stages, management is focused on reducing the progression through specific treatment modalities and drugs targeting the etiology, thereby halting the fibrosis [1]. The most accurate general examination of liver necrosis, inflammatory activity, and pathology for the prognosis of liver disease is now provided by a liver biopsy, which is a diagnostic test. Trauma, abnormal diseases, and bleeding are some of the other difficulties that restrict the use of liver biopsy [2]. The most recent prognostic modalities, known as aspartate aminotransferase to platelet ratio index (APRI) and FibroTest scoring systems, use biochemical indicators that are not directly correlated with fibrosis but use statistical approaches to predict the fibrosis stage.

Furthermore, several liver imaging modalities have demonstrated potential in identifying and staging liver fibrosis. Nonetheless, transient electrography, fibrospect, and fibrocheck have recently been used [3-7]. Several studies have demonstrated APRI as a very effective noninvasive diagnostic for identifying advanced liver fibrosis [8,9].

There are very few studies conducted worldwide to assess the severity and prognosis of liver fibrosis in cirrhotic individuals using noninvasive approaches. In several foreign nations, as well as in India, APRI is advised as a specific indicator for cirrhosis prognostication and is a more affordable option than liver biopsy [10-12]. Numerous studies assessed APRI's sensitivity and specificity as a diagnostic tool and concluded it was the most effective noninvasive method for identifying liver disease. According to retrospective research (n=320) by Angelo et al., APRI had an 80% accuracy rate for predicting liver-related poor outcomes in patients with non-alcoholic fatty liver disease (NAFLD) and a 63% accuracy rate for predicting death or liver transplantation [13]. Therefore, to determine the usefulness of APRI as a noninvasive marker for liver cirrhosis among the Indian population, we decided to conduct a study to evaluate the efficacy of APRI as a noninvasive prognostic marker for liver cirrhosis among patients with liver cirrhosis attending a tertiary care center in India.

## Materials and methods

Study design, setting, and study period

Among patients with liver illness who visited the Department of Medicine at the Datta Meghe Institute of Higher Education and Research, a teaching hospital with tertiary care situated in the remote Wardha District, we conducted a prospective observational study. The Institute's ethics committee gave its approval before the study was started. The corresponding approval letter is numbered DMIMS(DU)/IEC/2020-21/9285. Two years were dedicated to data collection, from December 2020 to November 2022.

Study participants

The study's inclusion criteria were all patients with alcoholic liver disease (ALD) or hepatitis B (HBV) and hepatitis C virus (HCV)-positive status, non-alcoholic steatohepatitis (NASH) who underwent ultrasonography at our center and were between the ages of 18 and 70. Patients with hepatocellular carcinoma, obese patients, individuals with increased chest wall fat, patients on mechanical ventilation, patients with heart failure, patients taking antiplatelets, and patients with any other fever illness affecting platelets were among the exclusion criteria.

Sample size

Using a 95% confidence level and a prevalence of 80%, the sample size of 200 was determined by applying the formula (𝑍𝛼2∗(p)∗(1−p)/𝑐2). About 100 cases with cirrhosis and 100 controls without cirrhosis were included in the calculation.

Study procedure

**Figure 1:**
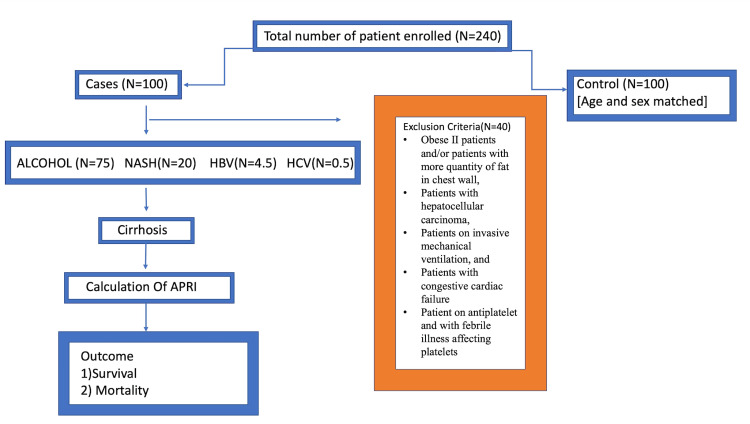
Flow chart of the study NASH: non-alcoholic steatohepatitis; HBV: hepatitis B virus; HCV: hepatitis C virus; APRI: aspartate aminotransferase to platelet ratio index Image by Dr. Harshitha Reddy

After receiving ethics permission from the Institute of Ethics Committee, the study was started. All the sociodemographic details, clinical features of the patients, and clinical findings such as prothrombin time (PT), liver function tests, kidney function tests, and total blood count, were recorded using a pretested semi-structured questionnaire. The patient outcome was considered in terms of survival and mortality correlated with APRI (APRI=(AST/upper limit of normal)×100/platelet count). Patient samples were collected under all aseptic precautions in a sodium citrate bulb, and the care diagnostics machine analyzed PT and international normalized ratio (INR) by using turbidimetry, and VITROS 5600 (Ortho Clinical Diagnostics, Raritan, NJ) was used to assess and estimate all parameters. The flow chart of the study is in Figure 1.

Data analysis

Data was imported into Excel (Microsoft Corporation, Redmond, Washington) and SPSS 20 (IBM, Chicago, Illinois) was used for analysis. For numerical data, mean ± standard deviation was employed, whereas frequency and proportions were utilized to summarize categorical variables. The chi-square test or Fischer exact test was used to compare the outcome variable with the independent variable. The area under the curve (AUC) was determined by Youden using the receiver operating characteristic (ROC) curve. A cutoff value for various parameters was employed in the method. The metrics of specificity, sensitivity, accuracy, negative predictive value (NPV), and positive predictive value (PPV) were computed to assess the efficacy of diagnostic devices. A p-value of less than 0.05 indicated statistical significance. 

## Results

Approximately 200 participants, 100 with cirrhosis and 100 with controls were gathered for our research. The study participants' demographics (ALD, NASH, or HBV, HCV virus), clinical traits, and laboratory values are shown in Table 1. Most study participants (47%) fell into the 40-60-year age range, with a mean age distribution of 48.3±13.6 years. A little over 87% had hemoglobin levels below 13 mg/dL, 37% had elevated red blood cell counts, and 60% had thrombocytopenia. Regarding activated partial thromboplastin time (aPTT) values, we found that almost 53% of the cases and controls had aPTT values between 29.5 and 35.0, and there was a large variability in these values. We observed that there was a significant association between APRI (p-value <0.001) and cases and controls, with higher APRI values observed among the cases (Table 1). 

**Table 1:** Characteristics and lab parameters of the study participants (N=200) APRI: aspartate aminotransferase to platelet ratio index; SGPT: serum glutamic pyruvic transaminase; SGOT: serum glutamic-oxaloacetic transaminase; INR: international normalized ratio; PT: prothrombin time; aPTT: activated partial thromboplastin time

Characteristics	Cases frequency (%)	Controls frequency (%)	P-value
Age group			
17–39 years	41 (67.2)	20 (32.8)	<0.0001
40-60 years	49 (51.6)	46 (48.4)	
>60 years	10 (22.7)	34 (77.3)	
Hb			
<13	83 (48.0)	90 (52.0)	0.66
13-17	15 (60.0)	46 (40.0)	
>17	2 (100.0)	0 (0.0)	
WBC			
<4000	23 (65.7)	12 (34.3)	0.06
4000-10000	36 (40.0)	54 (60.0)	
>10000	41 (54.7)	34 (45.3)	
Platelet counts			
<1.3	70 (58.8)	49 (41.2)	0.06
1.3–4.5	29 (36.7)	50 (63.3)	
>4.5	1 (50.0)	1 (50.0)	
aPTT			
<29.5	0 (0.0)	15 (100.0)	<0.0001
29.5–35.0	53 (49.5)	54 (50.5)	
>35.0	47 (60.3)	31 (39.7)	
PT			
11.9-13.5	13 (41.9)	18 (58.1)	0.32
>13.5	87 (51.5)	82 (48.5)	
INR			
0.8-1.0	5 (62.5)	3 (37.5)	0.47
>1.1	95 (49.5)	97 (50.5)	
SGOT			
15-46	18 (34.6)	34 (65.4)	0.009
>46	82 (55.4)	66 (44.6)	
SGPT			
7-50	59 (44.7)	73 (55.3)	0.03
>50	41 (60.3)	27 (39.7)	
ALP			
38-126	60 (46.5)	69 (53.5)	0.18
>126	40 (56.3)	31 (43.7)	
APRI			
<0.5	8 (24.2)	25 (75.8)	<0.0001
0.5-1.5	27 (40.9)	39 (59.1)	
>1.5	65 (64.4)	36 (35.6)	

APRI and its correlation with survival and non-survival (Table 2).

**Table 2:** APRI and outcomes APRI: aspartate aminotransferase to platelet ratio index

APRI	Survival	Non-survival	Total
<0.5	8	0	8
0.5-1.5	26	1	27
>1.5	57	8	65
Mean ± SD	5.47±9.57	6.77±4.77	

We observed that the most common factor associated with cirrhosis among the study participants was orthostatic hypertension (46%) followed by ascites (44%) (Table 3).

**Table 3:** Other associated factors with cirrhosis among the study participants (N=100)

Characteristic		Frequency (%)
Other associated factors of the patients		
Features of portal hypertension	46 (46.0)	
Ascites	44 (44.0)	
Encephalopathy	28 (28.0)	
Hepato-renal syndrome	18 (18.0)	
Dialysis	1 (1.0)	

The optimal cut-off value of APRI in discriminating cases from control was found to be 3.99 using the Youden method. Thus the sensitivity and specificity for APRI were observed to be 33.00% and 86.00%, respectively (Table 4).

**Table 4:** ROC analysis of APRI in detecting cirrhosis, N=200 APRI: aspartate aminotransferase to platelet ratio index; ROC: receiver operating characteristic

Cases	APRI		TOTAL
	+ (>3.99)	(≤3.99)	
+	33 (16.5%)	67 (33.5%)	100 (50.0%)
-	14 (7.0%)	86 (43.0%)	100 (50.0%)
TOTAL	47 (23.5%)	153 (76.5%)	200 (100.0%)

**Figure 2:**
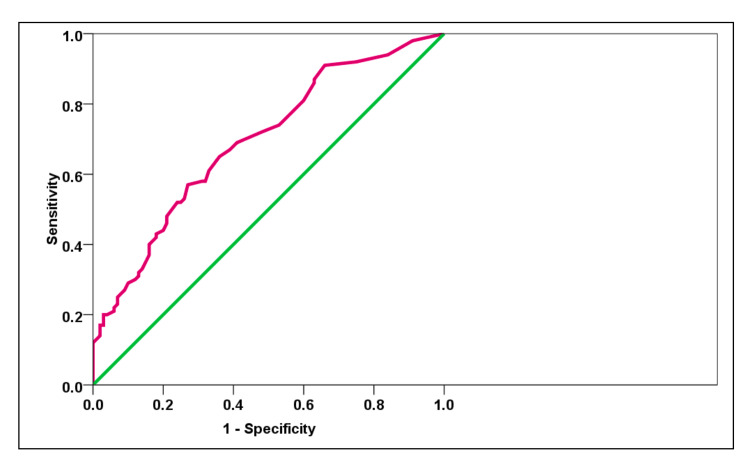
The area under the ROC curve for APRI in detecting cases was 0.693 APRI: aspartate aminotransferase to platelet ratio index; ROC: receiver operating characteristic Image by Dr. Harshitha Reddy

The PPV and NPV were observed to be 70.21% and 56.21%. We noted that the AUC of the ROC for APRI in detecting cases was 0.693 (Figure 2).

## Discussion

It is now recently documented that various studies have tried to address the shortcomings in the literature regarding the choice of an ideal noninvasive marker for ALD. Existent research in this field is also mainly focused on Western countries, and there needs to be more evidence from India, especially from the central part of India. Thus, we performed this cross-sectional analytical study to evaluate the effectiveness of APRI as a noninvasive prognostic marker for liver cirrhosis among patients with liver cirrhosis attending a tertiary care center in India. Our study findings showed that APRI is a vital tool for detecting cirrhosis.

In our study, we took around 200 cases, where 100 acted as controls and the remaining as cases. The age distribution showed that around 48% of the study participants belonged to the age groups of 40-60 years and that around 87% had hemoglobin values less than 13 mg/dL. These findings were observed to be similar to findings put forth by other studies done in various other study settings, such as Slaghi et al. and Maruyama et al., who estimated that the most commonly involved age group is middle age. Anemia is more prevalent among individuals with ALD [14,15].

Concerning the platelets and the WBC counts, we observed that around 38% had total counts of more than 10000 and that almost 3/5th had thrombocytopenia (counts <1.3 lakhs). These findings were also observed to be similar to findings observed by Ogasawara et al., who have also shown that ALD patients tend to have low platelet counts and neutrophilia (p-value of 0.03) [16]. These findings suggest rearrangements of platelet profiles due to variations in liver functioning. We also observed that around 40% of the patients had prolonged aPTT values, and around 4/5th of the participants showed prolongation of PT (>13.5 secs); these findings were observed to be similar to findings put forth by a study done by Slaghi et al. [14]. Cirrhosis is a condition that causes both coagulopathic and procoagulant conditions [17]. With platelet consumption, there are irregularities in platelet production as well as hypersplenism and reduced synthesis of clotting factors and anticoagulants that are reliant on and independent of vitamin K [18]. In people with cirrhosis, a lack of vitamin K-dependent clotting factors (II, VII, IX, and X) causes an increased international normalized ratio (INR). Although it is commonly believed that a raised INR indicates that a person is more likely to bleed, this is not the case for cirrhotic individuals, and the INR is less trustworthy than it would be in a person with normal liver function.

In our study, we observed that almost all (96%) cases had prolonged PT-INR, indicating the derangements in the clotting mechanism owing to liver cirrhosis. We also documented that there was a significant difference between the cases and controls concerning aPTT (p<0.0001). These findings were observed to be in line with other findings by Forkin et al. and Premkumar et al. [19,20].

Considering the liver function tests and its abnormalities, we observed that around 3/4th of the study participants were found to have increased SGOT (serum glutamic-oxaloacetic transaminase), and 1/3rd had an elevated SGPT (serum glutamic pyruvic transaminase). We also observed that there was a statistical difference between the distribution of these liver function parameters between the study groups, which was again demonstrated earlier by various other studies done by Sakka et al. and Albers et al. [21,22].

Concerning our main outcome of APRI estimation across the study groups, we observed that the mean distribution of APRI scores between cases and controls was 5.59±9.23 and 1.89±2.14, which was observed to be statistically significant (p-value of <0.001). This was observed to be in line with the findings followed by a study done by Castillo et al. and Snyder et al., who showed an elevation of APRI among cases with liver cirrhosis (p<0.0001) [23,24].

The mortality rates observed were 9% among the cirrhosis cases. A study by Lazo et al. has also shown that the mortality of cases with ALD varies between 3.1% and 16.1% [25]. When we tried comparing the proportion of APRI cases with values >1.5 across the survivors and non-survivors, we observed that the proportion of patients with APRI>1.5 was significantly higher in non-survival (88.9%) compared to survival (62.6%) (Z=4.30; p<0.0001). This was also comparable to the findings observed by Bakula et al. (p<0.0001) [26].

On comparing the validity of APRI scores in detecting cirrhosis, we observed that the AUC of the ROC for APRI in detecting cirrhosis was 0.693, and the optimal cutoff value of APRI was found to be 3.99 using the Youden method (p=0.0015). Using the same ROC analysis, we tried to obtain the other validation parameters, which showed a sensitivity, specificity, PPV, and NPV of 33%, 86%, 70%, and 56%, respectively. A study by Baranova et al. revealed that the APRI index had an AUC of 0.82 in detecting cirrhosis whereas in our study it was 0.693, which may be due to the small sample size. Therefore, based on the results of our investigation, we report that the APRI may be used as a noninvasive diagnostic for the identification of liver cirrhosis [27]. Ours was one of the very few studies that have correlated APRI with the severity of cirrhosis with a model for end-stage liver disease (MELD) and the Child-Pugh score was also one among the few studies that tried to compare APRI with various outcomes of cirrhosis in terms of mortality and morbidity from Indian settings, using all validated methods of investigations.

Limitations

The main limitation of the study is the APRI score distribution should be calculated in all liver disease etiologies and other demographic criteria. It could identify other case exceptions for using this score. The second limitation is the small sample size, which may have led to low ROC.

## Conclusions

Thus, our study results conclude that APRI can act as a useful noninvasive tool for prognostication and detection of liver cirrhosis. On comparing the validity of APRI scores in detecting cirrhosis, we observed that the AUC for APRI in detecting cirrhosis was 0.693, and the optimal cutoff value of APRI was 3.99. Furthermore, we encourage future research to comprehensively include the other noninvasive markers and compare them with APRI.
